# The English Dental Degree

**Published:** 1903-05-15

**Authors:** 


### THE ENGLISH DENTAL DEGREE.

The following questions show the scope of work required for the L.D.S. degree in the English colleges, and may be useful in comparison with American standards. These are the questions for 1902:

First Examination—Anatomy: 1. Describe in detail the development of the Maxilla and Mandible. Indicate the changes which these bones undergo during life.

2. Describe the attachments, relations, and actions of the right Ilio-psoas Muscle.

3. Give an account of the dissection necessary to expose the Popliteus Muscle from its origin to its insertion.

4. Describe the course, relations and vascular supply of the Œsophagus.

5. Describe the Hyoid Bone. Give the exact attachments of muscles and ligaments to it, and state what you know regarding its development and ossification.

6. Give the course and relations of the Deep Femoral Artery, and of its perforating branches.

7. Give the form, position and relations of the Transverse Colon, including its hepatic and splenic flexures.

8. Describe the Palmar Fascia. Give the relative position of the structures exposed on its removal.

Note.—Six of the eight questions on each subject must be answered.

Physiology: 1. Discuss the causes of the gaseous interchange in the Pulmonary and Systemic Capillaries. Give an account of the more important experiments which have been performed to decide the nature of the processes involved,

2. What nitrogenous substances occur in the Urine in addition to urea and uric acid? Discuss the chemical relationships and physiological importance of any two of these. How would you estimate the total nitrogen in a sample of Urine?

3. Describe the structure of the Organ of Corti. Explain the part played by the various structures of the Cochlea in the production of an Auditory Impulse.

4. Describe and contrast the neuro-muscular mechanism of the Œsophagus and the Small Intestine.

5. Describe the microscopical structure of the Pancreas. What is the composition of pancreatic juice, and how may the pure juice be obtained? What conditions modify the activity of pancreatic juice?

6. From what parts of the Cerebral Cortex may movements be excited? State the evidence that motor discharges from the central nervous system are rhythmic in character.

7. How may the intra-Cardiac pressure be investigated, and the time relations of the Cardiac Cycle determined?

8. Describe the manner by which changes in the size of the Pupil are brought about. What is the action of atropine, nicotine and pilocarpine on the neuro-muscular mechanism of the Iris?

Second Examination—Pathology, Therapeutics, and Surgery: 1. What are the Surgical complications, immediate and remote, which may arise in connection with Typhoid Fever? Discuss their pathology and treatment.

2. Discuss the aims, scope and limitations of Operations for Cancer of the Tongue.

3. Describe the varieties of Neuropathic Arthritis. Explain their pathology and give the chief diagnostic features of each.

4. What unusual conditions may be met with during the operation for Radical Cure of Inguinal Hernia? State how you would deal with them.

5. Enumerate the causes of Exophthalmos and discuss the prognosis in each case.

6. Describe the various forms of Tumors of the Urinary Bladder, and the treatment, operative and otherwise, which you would adopt.

7. Give an account of the pathoiogy and treatment of suppuration in the Mediastinum.

8. What are the predisposing and exciting causes of Aneurism in an extremity? How does a knowledge of these causes determine the treatment to be employed?

All questions must be answered.

Dental Metallurgy: 1. Describe the physical properties and preparation of Silver, its alloys as used for dental purposes, and the process of its assay by cupellation.

2. Describe the process of Nickel-plating.

3. How would you reduce pure Gold to a required carat? Describe the various processes by which plate gold of 18 carat may be prepared from scrap gold of several different and unknown carats.

4. What properties are required in Dies and Counter-dies? What metals are used for the purpose? Should simple metals or alloys be used, and why?

5. Give the physical properties and mode of preparation of Lead, and state the uses of lead and its alloys for dental purposes.

6. What qualities are requisite in an amalgam for dental purposes? Give a formula for a good dental amalgam, your reasons for the use of each ingredient, and the proportions in which each is beneficial.

7. What is meant by the hardness, malleablity, ductility, fusibility, and tenacity of a metal? Arrange any ten metals in their order with regard to these properties.

8. Describe the processes of coloring and gilding metals.

Anatomy and Physiology: 1. Describe the composition, sources and uses of mixed Saliva.

2. Give the origin, course and distribution of the Hypoglossal Nerve.

3. Give the origin, insertion and nerve-supply of the following muscles: Temporal, Superior Constrictor, Anterior Scalenus and Pectoralis Major.

Surgery and Pathology—5. Give the symptoms and treatment of impaction of a foreign body in the Œsophagus.

6. What are the symptons of Pyæmia? Explain how it may arise in connection with a wound.

7. Give the causes, symptoms and treatment of tubercular enlargement of the Cervical Lymphatic Glands.

Candidate should answer but four of the six questions.

Anatomy and Physiology: 1. What is the composition of normal Urine? How would you test for the presence of Albumen?

2. Give the relations of the Parotid Gland.

3. What Arteries supply the Scalp? Give their relative positions, and state from what trunk each arises.

Surgery and Pathology: 4. Give the causes, symptoms and treatment of blocking of Wharton's Duct.

5. What is meant by the terms Thrombosis and Embolism? Under what circumstances may each occur?

6. Give the causes, symptoms and possible complications of fracture of a Rib.

Dental Anatomy and Physiology: 1. In what respects do the teeth of Marsupials differ from those of other orders of Mammalia, and in what ways do they approach the dentitions of these different orders?

2. Having to prepare a microscopical section of a complete tooth germ before calcification has commenced:

(*a*) What age should the subject be?

(*b*) What part of the mouth would you select?

(*c*) How would you produce differential staining, and what colors would select each tissue?

(*d*) What could you demonstrate from such a section?

3 What are the different views held with regard to the calcification of Dentine? Describe them, and mention the arguments for and against them. Under what chemical conditions does the calcification of the dentine matrix occur?

Dental Surgery and Pathology: 4. Describe the treatment of exposed pulp in a temporary molar, and the treatment of sensitive cavities in temporary teeth without exposure.

5. What diseases may arise in the dental periosteum? Describe the pathology and treatment of each.

6. In extracting a tooth a small portion of the end of the root is broken and left in the socket. What pathological changes may arise? Give their symptoms and treatment.

Dental Anatomy and Physiology: 1. Describe the normal articulation of the teeth when the mouth is closed in (*a*) Man, (*b*) the Horse, (*c*) the Dog, and compare the movements of the teeth on one another during the act of mastication.

2. Describe the development of the first permanent Molar from the first appearance of the germ to the completion of the roots. From what portion of the tooth-band does it arise, and what inferences have been drawn from its mode of origin?

3. How would you prepare a section of a tooth to show (*a*) the structure of the Peridental Membrane, (*b*) the Nerves of the Pulp, (*c*) Interg.obular Spaces? What stains would you employ in each case?

Dental Surgery and Pathology: 1. What conditions of the teeth and mouth may lead to Necrosis of the Alveolus, and what is the appropriate treatment?

2. What are the chief causes of discoloration of a tooth, and what means may be employed to remedy the defect?

3. What conditions of the gums and teeth may give rise to fœtor of the breath? How should such cases be treated? Write two prescriptions for a suitable antiseptic and deodorant mouth-wash.

